# Adoption and impact of wearable healthcare devices on health outcomes among Malaysian tertiary students

**DOI:** 10.1177/20552076251386666

**Published:** 2025-12-25

**Authors:** Xiao Wei Tan, Siew Chin Ong, Christina Malini Christopher, Mohamed Hassan Elnaem, Mustapha Mohammed

**Affiliations:** 1 School of Pharmaceutical Sciences, 743742 Universiti Sains Malaysia, Pulau Pinang, Malaysia; 2 School of Pharmacy and Pharmaceutical Sciences, 2596 Ulster University, Coleraine, UK; 3 Biomedical Research Center, QU Health, Qatar University, Doha, Qatar

**Keywords:** Wearable healthcare devices, digital health, health literacy, quality of life, students

## Abstract

**Objective:**

The integration of wearable healthcare devices (e.g. smartwatches) into daily life has grown significantly worldwide, offering opportunities for continuous health monitoring and disease prevention. Despite their potential, the adoption of such technologies among younger populations, particularly students, remains underexplored. This study aims to explore the adoption and impact of wearable healthcare devices on health states and behaviors among students of tertiary institutions in Malaysia.

**Methods:**

A cross-sectional study was conducted among students across tertiary institutions in Malaysia from December 2022 to August 2023 using a validated self-administered questionnaire. Data collected from the study respondents include sociodemographic characteristics, digital technology usage, wearable healthcare device usage, eHealth literacy, and health-related quality of life. Multivariable logistic regression was used to identify factors associated with the adoption of wearable healthcare devices. A p ≤ 0.05 was considered statistically significant.

**Results:**

The study included 450 participants, with 80.2% using a wearable healthcare device. Most users were females (74%), under 20 years old (62.9%), and undergraduate students (75.6%). The findings showed a positive attitude toward the devices, with perceived usefulness (57.1%) being the main factor for adoption. Privacy concerns were significant, and health beliefs notably influenced adoption intentions. Participants had adequate eHealth literacy, with an average health-related quality of life score of 1.7 according to the EuroQol Five-dimension Three-level Questionnaire (EQ-5D-3L). The level of study significantly influenced adoption intention (p < 0.05), with pre-university students exhibiting higher inclinations toward adopting the devices.

**Conclusion:**

The study indicates that the functionality of these devices encourages tertiary students to adopt them for healthier lifestyles. However, students often lack confidence in identifying reliable health resources online, which can impact their ability to make informed health decisions.

## Background

In an era where healthcare is increasingly intertwined with technological advancements, the development of wearable healthcare devices has emerged as a transformative force. Wearable healthcare devices can track vital signs, including heart rate, body temperature, oxygen level, blood pressure, and blood glucose. The specificity of wearable healthcare devices enables the collection, monitoring, and analysis of users’ health information and exercise in real-time. In addition, the features of wearable healthcare devices are not limited to monitoring health parameters but also include mental status, stress level, workloads, and customized daily exercises. Wearable healthcare devices can empower patients to engage better in the health ecosystem and support personalized care delivery.^1^

A study on consumer acceptance and behaviors of wearable devices, such as smartwatches, is necessary because wearable healthcare technology is considered new to consumers, and understanding users’ continuous usage intentions is crucial.^2^ A previous study has shown that the user's attitude toward wearable devices increases with perceived usefulness, positively impacting their intention to use.^3^ The study reports that extending the technology acceptance model (TAM) to wearable smart clothing devices also proves that perceived usefulness is the key variable affecting consumer attitudes toward wearable devices. The study highlighted the perceived usefulness, perceived enjoyment, and social image impacting the intention to use wearable devices. With the invention of wearable technology, one of the essential features of wearable healthcare devices is to change the user's behavior and improve their health.^4^

On the other hand, wearable health devices are essential in advancing precision medicine by enabling the measurement of parameters showing health status.^5^ Wearable devices can also be used to relate physiological parameters to specific actions or movements, relevant for motion analysis and kinesiology. Wearable devices enable both resting-state health monitoring and the association of physiological data with specific movements, supporting detailed motion analysis and activity recognition.^6,7^ Such applications are crucial for precision medicine and rehabilitation contexts. The study conducted by Hsin in Taiwan reported that wearable devices can inspire users to engage in physical activity and that owners have better health outcomes than nonowners.^8^ Furthermore, the study by Lee et al. also indicated a positive relationship between the actual use of wearable health technology and the expectancy to improve health.^9^

Malaysia is considered a nation with low health consciousness, and it confronts a notable problem of high obesity rates among its adult population.^10,11^ Recently, initiatives have been embarked on to focus on harnessing wearable devices to monitor crucial health metrics such as heart rate, calorie expenditure, and physical activity levels.^12^ As part of this initiative, users can input their own data and enable remote monitoring by healthcare providers. While adopting wearable fitness trackers has brought advantages to society, there has been limited scholarly research on the factors influencing users’ intentions to adopt wearable healthcare devices. In addition, understanding Malaysian intentions to adopt wearable healthcare devices is crucial for the future success of the wearable health technology industry. It provides insights into developing strategies to enhance health outcomes.

The increasing healthcare costs have limited people's ability to cover medical expenses and take proactive steps to manage their health concerns. Hence, wearable fitness devices are crucial in helping users achieve wellbeing and provide personal convenience in their journey toward a healthier lifestyle. However, most of the studies focused on the technology attributes and the usefulness of wearable technologies. Amidst all these technological advancements in personalized healthcare delivery,^13,14^ there is a lack of studies being conducted on the factors affecting the usage of wearable devices among tertiary institution students in Malaysia. Factors influencing individuals “adoption intention and behaviour change in using these devices are limited, as wearable healthcare devices are still rising in Malaysia. It is necessary to categorize the features that move Malaysian users” behavioral intention and adoption of wearable devices.^15^ This study aimed to identify the key factors that motivate users to adopt health devices and how these devices influence their health states and behaviors.

## Methods

### Study design

A cross-sectional study was conducted among students of tertiary education in Malaysia using self-administered questionnaires from December 2022 to August 2023. The survey was designed to investigate the adoption and impact of wearable healthcare devices on health states and behaviors among the students in tertiary institutions in Malaysia. The study was conducted and reported according to the Strengthening the Reporting of Observational Studies in Epidemiology (STROBE) guideline.^16^

### Study participants

The study included adult student participants aged 18 years and above who are currently studying at any tertiary institution listed by the Ministry of Higher Education in Malaysia. The study excluded students on exchange programs.

### Sample size

The sample size of this study was calculated using Krejcie and Morgan's formula, with a 95% confidence level, and 80% study power.^17^ The calculated sample size was 385, based on the estimated population size of students of 1,325,699, across public and private tertiary institutions in Malaysia. A total of 450 students were finally recruited, considering possible missing data. This strategy aligns with best practices in survey research, which recommend oversampling by approximately 10% to 20% to ensure adequate data for analysis, even after handling incomplete or invalid responses.^18^

### Sampling method

Convenience sampling was utilized because it would be easier to access select participants from the target population.^19^ The target group of students in this study was from universities in Malaysia as listed by the Ministry of Higher Education Malaysia.

### Questionnaire tools

The questionnaire was administered in English, consisting of four major sections: (i) sociodemographic characteristics; (ii) adoption of wearable healthcare devices; (iii) health literacy; and (iv) health-related quality of life. The questionnaires utilized in this study were adapted from previous studies with minor language modifications to fit the context of this study, and were administered in English. The questionnaires were the Health Literacy toolkit (eHLA), the eHealth Literacy Questionnaire (eHLQ), and the EuroQol Five-dimension Three-level Questionnaire (EQ-5D-3L) ^4,20,21^

The questionnaire consisted of four (4) major sections, including (i) sociodemographic characteristics, (ii) adoption of wearable healthcare devices (perceived usefulness, perceived convenience, perceived irreplaceability, perceived credibility, health belief, adoption intention), (iii) eHealth literacy assessments, and (iv) health-related quality of life. The questionnaire comprised Likert scale questions and multiple-choice questions. The response options for “strongly agree” and “agree” were combined to create a single agreeable answer option. Similarly, the “strongly disagree” and “disagree” options were merged to form a single disagreeable answer option for analysis (Appendix 1).^22^

The validation of the questionnaire was conducted systematically to ensure its relevance and accuracy in the study context. Initially, existing scales from previous research were reviewed and adapted to align with the specific objectives of this study, with minor modifications in language to ensure cultural and contextual appropriateness for the Malaysian population. Two experts (MM and SCO) independently reviewed all the questionnaire items and confirmed that all items are relevant (content validity). The questionnaire was also shared with a sample (n = 20) of the target population to confirm the clarity of the items (face validity). The feedback from this pretest was used to refine the items. A pilot test was conducted, and Cronbach's alpha coefficients for adoption, eHealth literacy and health-related quality of life (HRQoL) (α=0.78, 0.80, 0.79, respectively) were calculated to confirm internal consistency.

### Data collection

A self-administered questionnaire was promoted using a convenience sampling method; invitations were sent to respondents through Facebook, WhatsApp, Instagram, and email. The data were collected from December 2022 to August 2023. Participants were provided with basic information, including the aims of the study and a statement ensuring the confidentiality of their information. The estimated average time required to complete the questionnaire was around 10 minutes.

### Data analysis

Data were analyzed using Statistical Packages for Social Sciences (SPSS) version 29. Descriptive statistics were used to summarize sociodemographic characteristics, digital device usage, adoption of wearable healthcare devices, as well as eHealth literacy and health-related quality of life. The Shapiro-Wilk test was used to check the normality of the continuous data. Multivariable regression analysis was used to determine the predictors of adopting wearable healthcare devices. A p ≤ 0.05 was considered statistically significant using the 95% confidence interval.

## Results

### Sociodemographic characteristics

The questionnaire was completed by 450 participants from various tertiary institutions across Malaysia, with the majority being females [n = 333 (74%)] and were between 18 and 20 years old [n = 283 (62.9%)]. The number of female respondents is higher, reflecting the current trend in Malaysia where female students outnumber males in tertiary education.^23^ A summary of the sociodemographic characteristics of participants is presented in Table 1.

**Table 1.** Sociodemographic characteristics of study participants.

Characteristics	Frequency (n)	Percentage
Gender		
Male	117	26.0
Female	333	74.0
Age group		
Median (interquartile range)		20
18–20 years	283	62.9
Over 21 years	167	37.1
Race		
Malay	274	60.9
Chinese	159	35.3
Indian and others	17	3.8
Region of origin		
East Coast	42	9.3
Northern	277	61.6
Central	66	14.7
Southern	49	10.9
East Peninsular	16	3.6
Type of institution		
Public	393	87.3
Private	57	12.7
Level of study		
Pre-university	89	19.8
Undergraduate	340	75.6
Postgraduate	21	4.7
Year of study		
Year 1	247	54.9
Year 2	111	24.6
Year 3	70	15.6
Year 4	22	4.9
Household income*		
Bottom 40% / B40	250	55.6
Middle 40% / M40	160	35.5
Top 20% / T20	40	8.9
Presence of chronic disease		
Yes	8	1.8
No	442	98.2

Keys: *Categories based on household income in Malaysia^22^ [Bottom 40% / B40 (< RM4850); Middle 40% / M40 (RM4850 – RM10959); Top 20% / T20 (≥ RM10960)]; N = 450.

### Usage of digital technology devices

The study results showed that most of the respondents always used computers [n = 222 (49.3%)], smartphones/tablets [n = 377 (83.8%)] and internet services [n = 376 (83.6%)] in their daily lives. However, a significant number never or rarely use wearable healthcare devices [n = 208 (46.2%)]. A summary of the usage of digital technology devices is presented in Table 2.

**Table 2.** Usage of digital technology devices.

Device	Frequency, n (%)				
	**Never**	**Rarely**	**Sometimes**	**Often**	**Always**
Computer (laptop/desktop)	2 (0.4)	14 (3.1)	50 (11.1)	162 (36.1)	222 (49.3)
Smartphone/tablet	1 (0.2)	4 (0.9)	8 (1.8)	60 (13.3)	377 (83.8)
Internet and IT services	1 (0.2)	5 (1.1)	13 (2.9)	55 (12.2)	376 (83.6)
Wearable healthcare device	89 (19.8)	119 (26.4)	134 (29.8)	57 (12.7)	51 (11.3)

Keys: N = 450.

IT: internet technology.

### Adoption of wearable healthcare devices

Regarding perceived usefulness, most of the participants agreed that wearable healthcare devices are useful for personal health management [n = 404 (89.8%)], helpful in developing healthy habits [n = 393 (87.4%)], and maintaining a healthy status [n = 390 (86.7%)]. Regarding perceived convenience, respondents generally find wearable healthcare devices easy to use, with over 85% agreeing that they are easy to learn [n = 385 (85.5%)], the information is clear, and understandable [n = 376 (83.6%)]. A significant portion also agrees that these devices are easy to carry [n = 403 (89.5%)] and have access to wearable healthcare devices anytime [n = 364 (80.9%)].

Furthermore, regarding perceived irreplaceability, a considerable portion agrees that wearable healthcare devices are superior to traditional devices [n = 277 (61.5%)]. Respondents largely recognize functional differences between wearable devices and traditional ones [n = 347 (77.2%)]. Regarding perceived credibility, the majority of the respondents find that data provided by wearable healthcare devices are in line with their personal health data [n = 366 (81.4%)], credible [n = 351 (78%)]. In addition, they reported being more likely to use the devices if their health data is protected [n = 398 (88.4%)], and were worried about the safety of the devices [n = 282 (62.6%)]. A summary of the adoption of wearable healthcare devices is presented in Appendix 2.

### Health beliefs on the adoption of wearable healthcare devices

Most of the respondents expressed a desire to change unhealthy habits and minimize potential health risks by incorporating wearable healthcare devices into their routines [n = 416 (92.4%)]. Additionally, a high percentage of respondents believe they can effectively improve their health status through various means such as sports, healthy eating, and practicing healthy lifestyles [n = 423 (94%)]. A summary of the health beliefs on the adoption of wearable healthcare devices is presented in Appendix 3.

### Adoption intention of wearable healthcare devices

Almost half of the participants agreed that they realized that bad living habits would cause harm to their health [n = 425 (94.5%)]. Most of the participants agreed and were interested in using the wearable healthcare device [n = 371 (82.4%)] and planned to adopt or continue to adopt the wearable healthcare device in the future [n = 362 (80.4%)]. A summary of the adoption intention of wearable healthcare devices is presented in Appendix 4.

### eHealth literacy assessments

Most of the participants demonstrated agreement regarding their ability to find, evaluate, and use health resources on the Internet. Majority of the participants know how to find helpful health resources on the Internet [n = 369 (82%)], what health resources are available on the Internet [n = 359 (79.8%), where to find helpful health resources on the Internet [n = 356 (79.1%)] and know how to use the health information find on the Internet to help them [n = 373 (82.9%)]. Furthermore, over 72% of the participants affirmed having the skills needed to evaluate the health resources found on the Internet to help them [n = 328 (72.9%)]. In addition, most of the participants indicated agreement with their capability to discern high-quality from low-quality health resources on the Internet [n = 294 (65.3%)] and feel confident in using information from the Internet to make health decisions [n = 310 (68.9%)]. A summary of the eHealth literacy assessments is presented in Table 3.

**Table 3.** eHealth literacy assessments.

Statement	Frequency, n (%)				
	**Strongly disagree**	**Disagree**	**Undecided**	**Agree**	**Strongly agree**
I know how to find helpful health resources on the Internet	3 (0.7)	20 (4.4)	58 (12.9)	241 (53.6)	128 (28.4)
I know what health resources are available on the Internet	3 (0.7)	13 (2.9)	75 (16.6)	242 (53.8)	117 (26)
I know where to find helpful health resources on the Internet	3 (0.7)	21 (4.7)	70 (15.5)	242 (53.8)	114 (25.3)
I know how to use the health information I find on the Internet to help me	2 (0.4)	20 (4.5)	55 (12.2)	256 (56.9)	117 (26)
I have the skills I need to evaluate the health resources I find on the Internet	2 (0.4)	25 (5.6)	95 (21.1)	230 (51.1)	98 (21.8)
I can tell high-quality from low-quality health resources on the Internet	5 (1.1)	30 (6.7)	121 (26.9)	208 (46.2)	86 (19.1)
I feel confident in using information from the Internet to make health decisions	5 (1.1)	31 (6.9)	104 (23.1)	218 (48.4)	92 (20.5)

Keys: N = 450.

### Health-related quality of life

Five statements regarding mobility, self-care, usual activities, pain/discomfort, and anxiety/depression were asked, and most respondents had no problems (interquartile range (IQR) = 1). Most of the respondents had no problem walking about [n = 422 (93.8%)], self-care [n = 423 (94%)], usual activities [n = 403 (89.6%)], pain or discomfort [n = 326 (72.4%)], and anxiety or depression [n = 272 (60.4%)]. In addition, the respondents rated their health as (median = 77.0, IQR = 1.7). The overall median of 1.0 means that at least half of your participants reported no health problems across all EQ-5D-3L domains. A summary of the HRQoL of users of wearable healthcare devices is presented in Table 4.

**Table 4.** Health-related quality of life using the EQ-5D-3L.

Statement	n (%)	Median (IQR)
Mobility		1
I have no problems walking about	422 (93.8)	
I have some problems walking about	14 (3.1)	
I am confined to bed	14 (3.1)	
Self-care		1
I have no problems with self-care	423 (94)	
I have some problems washing or dressing myself	26 (5.8)	
I am unable to wash or dress myself	1 (0.2)	
Usual activities (e.g. work, study, or leisure activities)		1
I have no problems with performing my usual activities	403 (89.6)	
I have some problems performing my usual activities	45 (10)	
I am unable to perform my usual activities	2 (0.4)	
Pain/discomfort		1
I have no pain or discomfort	326 (72.4)	
I have moderate pain or discomfort	117 (26)	
I have extreme discomfort	7 (1.6)	
Anxiety/depression		1
I am not anxious or depressed	272 (60.4)	
I am moderately anxious or depressed	162 (36)	
I am extremely anxious or depressed	16 (3.6)	
Overall health status [VAS 0–10], median (IQR)		77.0 (1.7)

Keys: N = 450.

EQ-5D-3L: EuroQol five-dimension three-level questionnaire; IQR: interquartile range; VAS: visual analog scale.

### Factor associated with the use of wearable healthcare devices using multivariable logistic regression

A multivariable logistic regression was conducted to study the effects of gender, age, race, type of institution, level of education, household income, presence of chronic disease, eHealth literacy score, and health-related quality of life score on the likelihood of use of a wearable healthcare device. Among the factors studied, only the level of education showed significant associations with the use of wearable healthcare devices (p ≤ 0.05). A summary of the results of multivariable logistic regression is presented in Table 5.

**Table 5.** Factors associated with the use of wearable healthcare devices using multivariable logistic regression.

Characteristics	SE	Wald	Significance	OR (95% CI)
Gender				
Male	0.230	0.419	0.517	1.16 (0.74–1.82)
Female (*Ref*)	−	−	−	−
Age (years)				
Under 20 years	0.205	0.782	0.377	0.83 (0.56–1.25)
Over 21 years (*Ref*)	−	−	−	−
Race				
Malay	0.522	0.028	0.866	1.09 (0.39–3.04)
Chinese	0.531	0.023	0.880	1.08 (0.38–3.07)
Indian and others (*Ref*)	−	−	−	−
Type of institution				
Public	0.315	1.178	0.278	1.41 (0.76–2.61)
Private (*Ref*)	−	−	−	−
Level of study				
Pre-university	0.613	8.017	0.005	5.68 (1.71–18.88)
Undergraduate	0.585	3.563	0.059	3.02 (0.96–9.49)
Postgraduate (*Ref*)	−	−	−	−
Household income				
Bottom 40% / B40	0.366	0.825	0.364	1.40 (0.68–2.86)
Middle 40% / M40	0.376	0.211	0.646	0.84 (0.40–1.76)
Top 20% / T20 (*Ref*)	−	−	−	−
Chronic disease				
Yes	0.842	2.671	0.102	0.25 (0.05–1.32)
No	−	−	−	−
eHealth literacy score	0.280	0.694	0.405	0.79 (0.46–1.37)
HRQoL score	0.251	3.058	0.080	1.55 (0.95–2.53)

Keys: Bottom 40% / B40 (< RM4850); Middle 40% / M40 (RM4850 – RM10959); Top 20% / T20 (≥ RM10960); n = 450.

CI: confidence interval; HRQoL: health-related quality of life; OR: odds ratio; Ref: reference group; SE: standard error.

## Discussion

Our study findings indicated a positive attitude toward wearable healthcare devices, with perceived usefulness emerging as the primary factor influencing the adoption. Participants’ health beliefs significantly influenced their adoption intention. Most respondents demonstrated adequate knowledge and skills related to eHealth literacy. Compared to previous research, this study's primary contribution lies in investigating the adoption intention and impact of wearable healthcare devices on health states and behaviors.^8,24^

The usage of wearable healthcare devices among students in Malaysia was slightly lower than that of adults in the United States.^25,26^ While privacy concerns were noted as one of the potential reasons for this hesitance, they were not the primary concern reported by the respondents.^26^ The findings were comparable with the studies in other countries, such as Hong Kong and the United States, where privacy issues were noted as the primary concern affecting the adoption intention.

Our findings regarding the adoption of wearable healthcare devices indicated that students in Malaysian tertiary institutions consider perceived usefulness, convenience, irreplaceability, and credibility as crucial factors influencing their adoption of these devices, aligned with previous research.^27^ This study suggested that the adoption intention of wearable healthcare devices was not solely dependent on perceived usefulness but also associated with consumers’ perceptions. In general, the results demonstrated that perceived usefulness played a primary role in influencing Malaysian tertiary students to adopt wearable technology. This finding further deepens the conclusion obtained from existing studies that wearable healthcare devices can enhance user perception of health and assist them in achieving their goals in improving health outcomes.^28^

Besides, addressing concerns related to credibility and safety was crucial to foster a greater adoption and sustained usage among users. The result of this study was aligned with a study on young adults in Croatia, which highlighted that young adults found that wearable technology brings benefits to them. Still, privacy concerns, such as possibilities of access and theft of personal data, and the impossibility of permanent erasure of personal data, may restrict their interest in using it.^29^ However, their intention to adopt may decrease if privacy concerns are not addressed. Hence, further action should emphasize addressing these issues to enhance user trust and acceptance.

Additionally, this study also revealed that health beliefs significantly influence students’ intention to use these devices. Health belief refers to an individual's perceptions and attitudes regarding their susceptibility to illness, the severity of potential health issues, and the effectiveness of preventive actions.^30^ This result further confirmed the study done in Hong Kong, which demonstrated that health beliefs are significantly impacting the adoption intention of wearable healthcare technology.^31^ In other words, students with a higher level of health consciousness tend to adopt wearable healthcare devices to improve their health status.

The results of this study indicated that most tertiary students possess knowledge and skills related to digital health literacy. Malaysian tertiary institution students were proficient at seeking online health information from various sources. Although participants were proficient in navigating online health resources, many lacked confidence in critically evaluating their quality, highlighting the need for improved digital health literacy training programs in educational curricula. Many participants likely possess strong technical skills, which enable them to engage with health information online and participate in eHealth activities effectively. However, when it comes to the critical evaluation aspect of eHealth literacy, participants may struggle to discern high-quality, evidence-based health resources from unreliable or misleading ones. This could be due to limited experience in assessing the credibility of sources, recognizing signs of misinformation, or differentiating between authoritative and nonauthoritative platforms.^32^

Furthermore, the examination of the health-related quality of life among users of wearable health devices revealed that students do not face issues in mobility, self-care, usual activities, pain/discomfort, and anxiety/depression. From our results, students had an overall median health-related quality of life score of one based on the EQ-5D-3L study. The median EQ-5D-3L index score was 1.0, indicating that at least half of the respondents reported being in full health across all measured domains. Still, overall, the population maintained a good level of perceived health of 77%. This assessment of health-related quality can serve as a guideline for investigating the adoption intention of wearable healthcare devices and understanding their impact on student behaviors.^33^ Besides, understanding the current health-related quality of life can help in tailoring the wearable healthcare device features to meet the needs and preferences of students better.

Additionally, this study revealed that the level of education significantly influences the intention to adopt wearable healthcare devices. Specifically, pre-university students demonstrated a higher likelihood of adopting these devices compared to those at higher academic levels. This trend suggests that the appealing designs and features of wearable healthcare devices may resonate more with students at these earlier stages of their education, which aligns with the findings of Dehghani ^2^. Additionally, this population appears to be aligned with current technology and stays updated with the latest devices. The adoption rate of using devices is very likely higher in this age group compared to others.^34^

### Strengths and limitations

Firstly, this study's strength lies in its narrow and homogeneous focus on tertiary institution students. The research clearly identifies factors that influence the use of wearable devices, providing valuable insights for stakeholders interested in expanding access to these technologies. However, generalizability may be limited as the study samples were restricted to students only. The findings of this study are unique as they focused on a Malaysian educational institution, where students are exposed to a multicultural and multilingual learning environment. Such diversity may influence the use of wearable devices through various learning preferences and adaptability in ways that differ from more homogenous student populations elsewhere. Including students from secondary education institutions could yield more precise insights, as younger generations are increasingly adopting wearable devices.

The use of a convenience sampling method may have introduced selection bias, as the sample primarily consisted of students from tertiary institutions, potentially limiting the applicability of the results to broader populations. The convenience sampling method employed could result in constraints on the generalizability of the findings. Additionally, the reliance on self-reported data may be subject to recall and social desirability biases, which could influence the accuracy of the responses. Furthermore, the exclusion of individuals with chronic diseases or specific health conditions may have led to an underrepresentation of participants who might have different health beliefs or wearable device usage patterns. Furthermore, it is expected that different factors may influence a person's intention to use wearable healthcare devices, given the variety of wearable healthcare devices available, such as type and size, which may have notable effects on user experience.

### Implication for practice

The study findings suggested several implications for policymakers, healthcare providers, and device manufacturers. For policymakers, the adoption of wearable healthcare devices among students could be supported through financial incentives, such as subsidies or tax breaks, making these technologies more affordable and accessible. Additionally, addressing the data privacy issue is essential to protect personal health information collected by these devices, ensuring student users feel secure. It is essential to optimize the technical attributes to enhance usability and to align with individual preferences. For device manufacturers, it is crucial to design wearables that are both user-friendly and affordable for students. The device's function should be improved in terms of usefulness, data accuracy, and credibility to enhance adoption and effectiveness further. For healthcare providers, the integration of wearable health data into routine medical care could significantly enhance patient monitoring and care management. Moreover, wearable data can be used to identify students at risk for health issues, such as physical inactivity or sleep disturbances, allowing for timely, targeted interventions that address these concerns proactively. The study findings could guide the promotion of more effective integration of wearable devices to support student health and wellbeing.

## Conclusion

This study provided valuable insights into the adoption of wearable healthcare devices among students of tertiary institutions in Malaysia. Practically, the study highlights the most critical factors, such as the functionality of the technology and privacy concerns, that influence students’ adoption of wearable devices. Future research should consider exploring a wider variety of user demographics, including individuals with specific health conditions.

## Supplemental Material

sj-docx-1-dhj-10.1177_20552076251386666 - Supplemental material for Adoption and impact of wearable healthcare devices on health outcomes among Malaysian tertiary students Supplemental material, sj-docx-1-dhj-10.1177_20552076251386666 for Adoption and impact of wearable healthcare devices on health outcomes among Malaysian tertiary students by Xiao Wei Tan, Siew Chin Ong, Christina Malini Christopher, Mohamed Hassan Elnaem and Mustapha Mohammed in DIGITAL HEALTH

## References

[1] N. B . What is the future of wearable technology in healthcare? United States: College of Medicine Blog Network, 2021.

[2] Dehghani M . Exploring the motivational factors on continuous usage intention of smartwatches among actual users. Behav Inf Technol 2018; 37: 145–158.

[3] Chae J-M . Consumer acceptance model of smart clothing according to innovation. Int J Human Ecol 2009; 10: 23–33.

[4] Zhang M Luo M Nie R , et al. Technical attributes, health attribute, consumer attributes and their roles in adoption intention of healthcare wearable technology. Int J Med Inf 2017; 108: 97–109. 10.1016/j.ijmedinf.2017.09.016 29132639

[5] cheol Jeong I Bychkov D Searson PC . Wearable devices for precision medicine and health state monitoring. IEEE Trans Biomed Eng 2018; 66: 1242–1258. 10.1109/TBME.2018.2871638 31021744

[6] Francia C Donno L Rodriguez MC , et al. Real-time monitoring of physiological and postural parameters to evaluate human reactions in virtual reality for safety training. Sensors (Basel, Switzerland) 2025; 25: 4400. 40732528 10.3390/s25144400 PMC12298619

[7] Iosa M Picerno P Paolucci S , et al. Wearable inertial sensors for human movement analysis. Expert Rev Med Devices 2016; 13: 641–659. 27309490 10.1080/17434440.2016.1198694

[8] Yen H-Y Huang H-Y . Comparisons of physical activity and sedentary behavior between owners and non-owners of commercial wearable devices. Perspect Public Health 2021; 141: 89–96. 33733947 10.1177/1757913921989389

[9] Lee SM Lee D . Healthcare wearable devices: an analysis of key factors for continuous use intention. Service Business 2020; 14: 503–531.

[10] Mamun AA Hayat N Zainol NRB . Healthy eating determinants: a study among Malaysian young adults. Foods 2020; 9: 974. 32717851 10.3390/foods9080974 PMC7466180

[11] Bernama . Unhealthy nation with low health awareness. Malaysia: New Strait Times, 2021. https://www.nst.com.my/news/nation/2021/11/744515/malaysia-unhealthy-nation-lowhealth-awareness (accessed 3rd April 2025).

[12] Basu M. 2016. New health records and remote monitoring to be launched. https://govinsider.asia/intl-en/article/exclusive-malaysias-plan-to-overhaul-healthcare.

[13] Andersson P Mattsson L-G . Service innovations enabled by the “internet of things”. Imp Journal 2015; 9: 85–106.

[14] Atzori L Iera A Morabito G . The internet of things: a survey. Comput Netw 2010; 54: 2787–2805.

[15] Hayat N Salameh AA Mamun AA , et al. Exploring the mass adoption potential of wearable fitness devices in Malaysia. Digit Health 2023; 9: 20552076231180728. 20230604. 37325073 10.1177/20552076231180728 PMC10265353

[16] Hess DR . Observational studies. Respir Care 2023; 68: 1585–1597. 37339891 10.4187/respcare.11170 PMC10589119

[17] Krejcie R Morgan D . Determining sample size for research activities. Educ Psychol Measur 1970; 30: 607–610.

[18] Memon MA Ting H Cheah J-H , et al. Sample size for survey research: review and recommendations. J Appl Struct Equat Model 2020; 4: i–xx.

[19] Elfil M Negida A . Sampling methods in Clinical Research; an educational review. Emerg (Tehran) 2017; 5: e52. 20170114. PMC5325924 28286859

[20] Group TE . EuroQol-a new facility for the measurement of health-related quality of life. Health Policy 1990; 16: 199–208. 10109801 10.1016/0168-8510(90)90421-9

[21] Norman CD Skinner HA . eHEALS: the eHealth literacy scale. J Med Internet Res 2006; 8: e507. 10.2196/jmir.8.4.e27 PMC1794004 17213046

[22] Ooi C . The T20, M40 and B40 income classifications in Malaysia. 2017. 2018.

[23] Elhadary Y Samat N . Addressing gender disparity in public universities of Malaysia: challenges and achievements. J Higher Educ Theory Pract 2023; 23: 156–172.

[24] Lee J Kim D Ryoo H-Y , et al. Sustainable wearables: wearable technology for enhancing the quality of human life. Sustainability 2016; 8: 466.

[25] Chandrasekaran R Khattula V Moustakas E . Use of wearable healthcare devices by US adults: patterns of use and key predictors. JMIR 2020; 22: (1). 10.2196/22443 PMC7600024 33064083

[26] Kapoor V Singh R Reddy R , et al. Privacy issues in wearable technology: an intrinsic review. In: Proceedings of the International conference on innovative computing & communications (ICICC), India, 2020, pp. 1.

[27] Singh N Misra R Singh S , et al. Assessing the factors that influence the adoption of healthcare wearables by the older population using an extended PMT model. Technol Soc 2022; 71: 102126.

[28] Choudhury A Asan O . Impact of using wearable devices on psychological distress: analysis of the health information national trends survey. Int J Med Inf 2021; 156: 104612. 10.1016/j.ijmedinf.2021.104612 34649113

[29] Papić A Ðurdević K . Young adults’ privacy concerns about wearable technology. In: 2021 44th International convention on information, communication and electronic technology (MIPRO). Croatia: IEEE, 2021, pp.537–541.

[30] Daniati N Widjaja G Olalla Gracìa M , et al. The health belief model’s application in the development of health behaviors. Health Educ Health Promot 2021; 9: 521–527.

[31] Chau KY Lam MHS Cheung ML , et al. Smart technology for healthcare: exploring the antecedents of adoption intention of healthcare wearable technology. Health Psychol Res 2019; 7: 8099. 31583292 10.4081/hpr.2019.8099 PMC6767842

[32] Allen-Meares P Lowry B Estrella ML , et al. Health literacy barriers in the health care system: barriers and opportunities for the profession. Health Soc Work 2020; 45: 62–64. 31993624 10.1093/hsw/hlz034 PMC8453407

[33] Wang L Lu Y Dai Z , et al. Obtaining EQ-5D-3L utility index from the health status scale of traditional Chinese medicine (TCM-HSS) based on a mapping study. Health Qual Life Outcomes 2022; 20: 64. 36522665 10.1186/s12955-022-02076-9 PMC9753309

[34] Kyytsönen M Vehko T Anttila H , et al. Factors associated with use of wearable technology to support activity, well-being, or a healthy lifestyle in the adult population and among older adults. PLOS Digital Health 2023; 2: e0000245. 10.1371/journal.pdig.0000245 PMC10171588 37163490

