# Supplementary material for: Adoption and impact of wearable healthcare devices on health outcomes among Malaysian tertiary students
Source: Digit Health. 2025 Dec 25;11:20552076251386666. doi: 10.1177/20552076251386666 (PMC12745509; doi:10.1177/20552076251386666)
Supplement: sj-docx-1-dhj-10.1177_20552076251386666 - Supplemental material for Adoption and impact of wearable healthcare devices on health outcomes among Malaysian tertiary students [file sj-docx-1-dhj-10.1177_20552076251386666.docx]

**Appendix 1 Questionnaire**

**Adoption and impact of wearable healthcare devices on health states and behaviours among students of tertiary institutions in Malaysia**

**Section A: Socio-demographic characteristics**

Gender*

- Male
- Female

Age*

Race*

- Malay
- Chinese
- Indian
- Other:

State of origin*

- Johor
- Kedah
- Kelantan
- Malacca
- Negeri Sembilan
- Pahang
- Penang
- Perak
- Perlis
- Sabah
- Sarawak
- Terengganu
- Selangor
- Kuala Lumpur
- Labuan
- Putrajaya

Institution name*

Type of institution*

- Public
- Private
- Other

Level of study*

- College/Pre-university/Diploma
- Undergraduate
- Postgraduate

Course of study*

Year of study*

- Year 1
- Year 2
- Year 3
- Year 4
- Other

Household income*

- < RM4850 (Bottom 40% / B40)
- RM4850 – RM10959 (Middle 40% / M40)
- ≥ RM10960 (Top 20% / T20)

Presence of chronic disease*

- Yes
- No

Choose the option that best describes your usage on these devices.*

|  | Never | Rarely | Sometimes | Often | Always |
| --- | --- | --- | --- | --- | --- |
| Computer/Laptop/Desktop |  |  |  |  |  |
| Smartphone/tablet |  |  |  |  |  |
| The Internet and IT |  |  |  |  |  |
| Wearable healthcare device |  |  |  |  |  |
| Willingness to share the wearable  healthcare data |  |  |  |  |  |

**Section B: Perceived usefulness**

Choose the option that best describes your perception on the use of wearable healthcare device.*

|  | Strongly Disagree | Disagree | Undecided | Agree | Strongly Agree |
| --- | --- | --- | --- | --- | --- |
| It would be useful in my personal health  management. |  |  |  |  |  |
| It would help me to  develop healthy habits. |  |  |  |  |  |
| It would help me to maintain healthy status. |  |  |  |  |  |

**Section C: Perceived convenience**

Choose the option that best describes your perception on the use of wearable healthcare devices.*

|  | Strongly  Disagree | Disagree | Undecided | Agree | Strongly  Agree |
| --- | --- | --- | --- | --- | --- |
| Learning to use the device  would be easy for me. |  |  |  |  |  |
| The information showed is clear and understandable. |  |  |  |  |  |
| I think the device is easy to carry. |  |  |  |  |  |
| I have access to the device anytime. |  |  |  |  |  |

**Section D: Perceived irreplaceability**

Choose the option that best describes your perception on the use of wearable healthcare devices.*

|  | Strongly  Disagree | Disagree | Undecided | Agree | Strongly  Agree |
| --- | --- | --- | --- | --- | --- |
| I think the device are  superior to similar traditional devices. |  |  |  |  |  |
| I think there are some functional similar and differences between the  device and traditional devices. |  |  |  |  |  |

**Section E: Perceived credibility**

Choose the option that best describes your perception on the use of wearable healthcare devices.*

|  | Strongly  Disagree | Disagree | Undecided | Agree | Strongly  Agree |
| --- | --- | --- | --- | --- | --- |
| The data provided are in  line with my personal health data. |  |  |  |  |  |
| The software system is credible. |  |  |  |  |  |
| It is more possible for me to use it if my personal health information will be  protected. |  |  |  |  |  |
| I am worried about the safety of the device. |  |  |  |  |  |

**Section F: Health belief**

Choose the option that best describes your health belief.*

|  | Strongly Disagree | Disagree | Undecided | Agree | Strongly Agree |
| --- | --- | --- | --- | --- | --- |
| I hope I can change my  bad habits and thus  minimize damage to health. |  |  |  |  |  |
| I think I can improve my health status effectively in many ways like sports, eat healthy and practice  healthy lifestyle. |  |  |  |  |  |

**Section G: Adoption intention**

Choose the option that best describes your intention on the use of wearable healthcare device.*

|  | Strongly Disagree | Disagree | Undecided | Agree | Strongly Agree |
| --- | --- | --- | --- | --- | --- |
| I realize that bad living  habits will cause harm to  my health. |  |  |  |  |  |
| I am interested in using the wearable healthcare device. |  |  |  |  |  |
| I plan to adopt or continue to adopt the wearable healthcare device in the  future. |  |  |  |  |  |

**Section H: eHealth literacy assessments**

Choose the option that best describes your eHealth literacy.*

|  | Strongly Disagree | Disagree | Undecided | Agree | Strongly Agree |
| --- | --- | --- | --- | --- | --- |
| I know how to find helpful health resources  on the Internet. |  |  |  |  |  |
| I know what health  resources are available on the Internet. |  |  |  |  |  |
| I know where to find  helpful health resources on the Internet. |  |  |  |  |  |
| I know how to use the  health information I find  on the Internet to help me. |  |  |  |  |  |
| I have the skills I need to evaluate the health resources I find on the  Internet. |  |  |  |  |  |
| I can tell high quality  from low quality health  resources on the Internet. |  |  |  |  |  |
| I feel confident in using information from the Internet to make health  decisions. |  |  |  |  |  |

**Section I: Health-Related Quality of life of participants using EQ-5D-3L** Under each heading, please tick ONE box that best describes your health TODAY. Mobility

- I have no problems in walking about.
- I have some problems in walking about.
- I am confined to bed.

Self-Care

- I have no problems with self-care.
- I have some problems washing or dressing myself.
- I am unable to wash or dress myself.

Usual activities (eg. work, study, housework, family or leisure activities)

- I have no problems with performing my usual activities.
- I have some problems with performing my usual activities.
- I am unable to perform my usual activities.

Pain/Discomfort

- I have no pain or discomfort.
- I have moderate pain or discomfort.
- I have extreme of discomfort.

Anxiety/Depression

- I am not anxious or depressed.
- I am moderately anxious or depressed.
- I am extremely anxious or depressed.

Your health today (Choose from the range 0 (worst) to 10 (best)

0 1 2 3 4 5 6 7 8 9 10

Worst
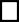

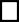

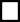

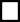

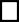

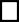

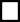

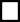

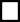

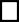

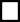
 Best

**Appendix 2** Adoption of wearable healthcare devices

| **Statements on the device** | **n (%)** | | | | |
| --- | --- | --- | --- | --- | --- |
|  | **Strongly Disagree** | **Disagree** | **Undecided** | **Agree** | **Strongly Agree** |
| **Perceived usefulness** | | | | | |
| Useful to my personal health management. | 7 (1.6) | 4 (0.9) | 35 (7.8) | 257 (57.1) | 147 (32.7) |
| Helpful to develop healthy habits. | 7 (1.6) | 7 (1.6) | 43 (9.6) | 237 (52.7) | 156 (34.7) |
| Helpful to maintain healthy status. | 8 (1.8) | 9 (2.0) | 43 (9.6) | 234 (52.0) | 156 (34.7) |
| **Perceived convenience** | | | | | |
| Easy to learn how to use. | 6 (1.3) | 8 (1.8) | 51 (11.3) | 240 (53.3) | 145 (32.2) |
| Information showed is clear and understandable. | 5 (1.1) | 8 (1.8) | 61 (13.6) | 238 (52.9) | 138 (30.7) |
| Easy to carry. | 6 (1.3) | 6 (1.3) | 35 (7.8) | 227 (50.4) | 176 (39.1) |
| I have access anytime. | 7 (1.6) | 17 (3.8) | 62 (13.8) | 215 (47.8) | 149 (33.1) |
| **Perceived irreplaceability** | | | | | |
| Are superior to similar traditional devices. | 10 (2.2) | 29 (6.4) | 134 (29.8) | 199 (44.2) | 78 (17.3) |
| Functional differences with the traditional devices. | 5 (1.1) | 14 (3.1) | 84 (18.7) | 241 (53.6) | 106 (23.6) |
| **Perceived credibility** | | | | | |
| Data provided are in line with my personal health data. | 5 (1.1) | 9 (2.0) | 70 (15.6) | 260 (57.8) | 106 (23.6) |
| They are credible. | 4 (0.9) | 13 (2.9) | 82 (18.2) | 255 (56.7) | 96 (21.3) |
| More possible to use if my personal health data will be protected. | 3 (0.7) | 9 (2.0) | 40 (8.9) | 253 (56.2) | 145 (32.2) |
| Worried about the safety of the device. | 13 (2.9) | 48 (10.7) | 107 (23.8) | 191 (42.4) | 91 (20.2) |
| Keys: N=450 |  |  |  |  |  |

**Appendix 3** Health belief on the adoption of wearable healthcare devices

| **Statements on the device** | **n (%)** | | | | |
| --- | --- | --- | --- | --- | --- |
|  | **Strongly Disagree** | **Disagree** | **Undecided** | **Agree** | **Strongly Agree** |
| **Health belief** | | | | | |
| I hope I can change my bad habits and thus minimize damage to my health. | 3 (0.7) | 0 (0) | 31 (6.9) | 213 (47.3) | 203 (45.1) |
| I think I can improve my health status effectively in many ways like sports, eating healthy and practice healthy lifestyle. | 3 (0.7) | 1 (0.2) | 23 (5.1) | 190 (42.2) | 233 (51.8) |
| **Keys:** N=450 |  |  |  |  |  |

**Appendix 4**  Adoption intention of wearable healthcare devices.

| **Statement** | **Frequency, n (%)** | | | | |
| --- | --- | --- | --- | --- | --- |
|  | **Strongly Disagree** | **Disagree** | **Undecided** | **Agree** | **Strongly Agree** |
| **Adoption intention** | | | | | |
| I realize that bad living habits will cause harm to my health. | 2 (0.4) | 3 (0.7) | 20 (4.4) | 201 (44.7) | 224 (49.8) |
| I am interested in using the wearable healthcare device. | 5 (1.1) | 7 (1.6) | 67 (14.9) | 222 (49.3) | 149 (33.1) |
| I plan to adopt or continue adopt the wearable healthcare device in the future. | 3 (0.7) | 6 (1.3) | 79 (17.6) | 222 (49.3) | 140 (31.1) |
| **Keys:** N=450 |  |  |  |  |  |
